# On‐deck pH measurement with integrated feedback control on a micro‐scale platform for monoclonal antibody low pH viral inactivation

**DOI:** 10.1002/btpr.88516

**Published:** 2026-05-05

**Authors:** Paras Sharma, Nikolas von den Eichen, Sabine Schweisgut, Lars Robbel, Michael Schmitt, Daniel G. Bracewell

**Affiliations:** ^1^ Department of Biochemical Engineering University College London London UK; ^2^ Biopharmaceutical Product Development CSL Innovation Marburg Germany

**Keywords:** downstream processing, high throughput process development, micro‐scale, monoclonal antibodies, scale‐down

## Abstract

The increasing demand for efficient monoclonal antibody manufacturing has accelerated the adoption of high throughput process development (HTPD) platforms, which enable rapid, automated screening of downstream operations. However, the integration of non‐chromatographic steps such as low pH viral inactivation (VI) within automated workflows remains limited, largely due to the absence of a micro‐scale method for accurate, on‐deck pH measurement and control. This study presents the development and implementation of an automated pH measurement and feedback control system using optical pH sensors immobilized within 96‐well microplates. The approach enables non‐invasive, real‐time monitoring of pH across the acidic range required for VI and is fully compatible with standard liquid‐handling platforms. Integration of a feedback control algorithm allowed autonomous acid and base addition to achieve precise target pH values during both acidification and neutralization phases. The method achieved strong agreement between measured and expected pH values following optimization of measurement conditions, including ionic strength adjustment. The system was further integrated with Sartobind® Q and cation exchange chromatography steps to demonstrate an end‐to‐end automated workflow. Systematic assessment of cation exchange chromatography performance under controlled loading conditions enabled direct visualization of separation behavior and early identification of sub‐optimal operating regions, demonstrating the platform's capability to expand experimental space and accelerate mechanistic process understanding. This work establishes a micro‐scale, fully automated downstream platform with pH control, bridging a critical technological gap and advancing the vision of an end‐to‐end HTPD system for biopharmaceutical purification.

## Graphical abstract

Integrating an automated, on‐deck pH measurement method with feedback control enables precise, real‐time pH adjustment during low pH viral inactivation on a micro‐scale platform.

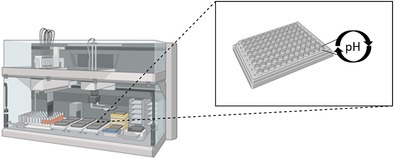

## 1 INTRODUCTION

The continued clinical and commercial success of monoclonal antibodies (mAbs) has driven the biopharmaceutical industry toward developing manufacturing processes that are both efficient and scalable. Improvements in upstream productivity over the past two decades have significantly increased cell culture titers, shifting the process bottleneck toward downstream purification.^1^ As a result, the need for accelerated, resource‐efficient process development in downstream operations has intensified. High throughput process development (HTPD) has emerged as a key enabler of this acceleration, providing a platform‐based approach that leverages miniaturization, automation, and parallelization to enhance experimental throughput and process understanding.^2^ These tools have demonstrated substantial value for chromatographic unit operations such as protein A affinity capture, ion exchange chromatography (IEX), and hydrophobic interaction chromatography (HIC), where workflows can be effectively translated from laboratory‐scale to micro‐scale while preserving process performance and scalability.^3^, ^4^, ^5^, ^6^

**FIGURE 1:**
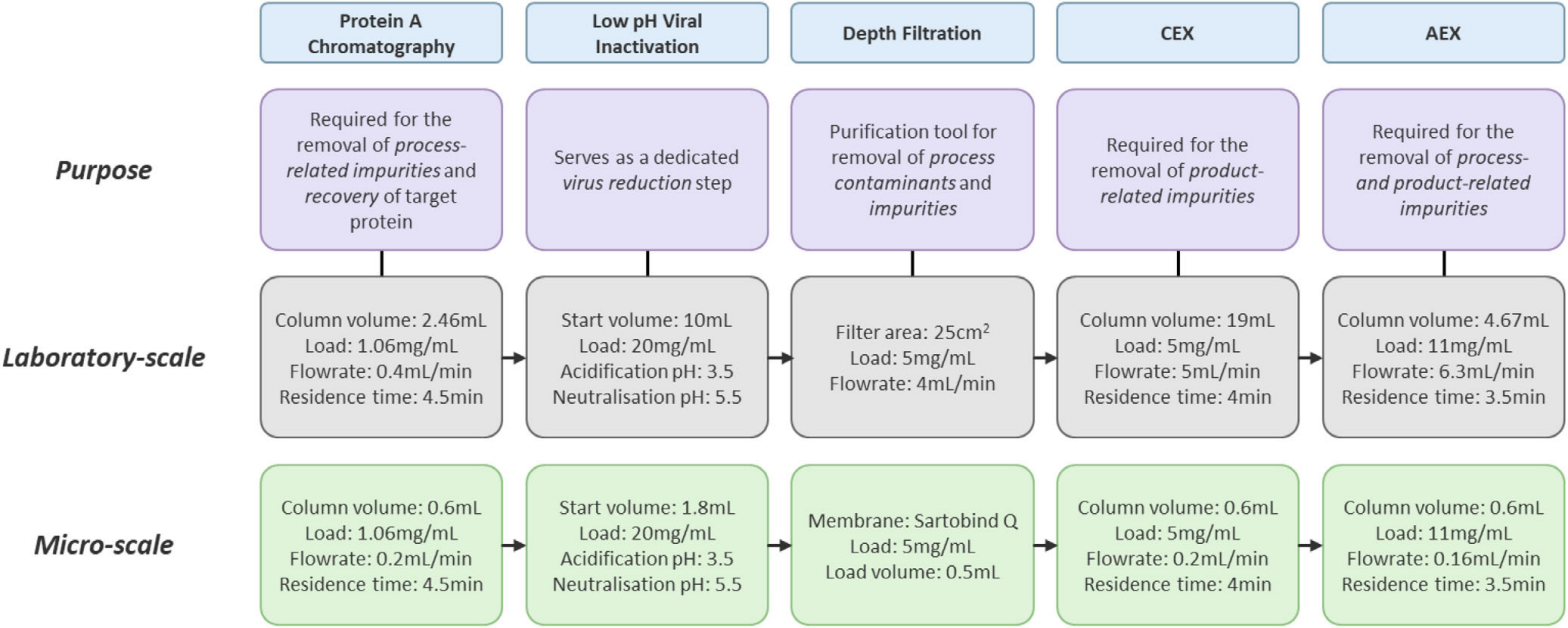
Process flowsheet of an exemplary and industry‐standard monoclonal antibody downstream process established on the micro‐scale platform. The purification sequence highlighted in gray and green represent the laboratory‐scale and micro‐scale, respectively, with the specified process parameters for each unit operation. The objective of each operation is also presented, emphasizing its role and significance within the overall purification sequence.

Despite these advances, the integration of non‐chromatographic operations within automated HTPD workflows remains limited. Operations such as low pH viral inactivation (VI) and depth filtration are integral to the mAb purification sequence but are often executed manually or off‐deck. This limitation hinders the ability to investigate interdependencies between unit operations and restricts overall process automation. Our previous studies have demonstrated the translation of protein A chromatography and low pH VI to the micro‐scale, which was subsequently integrated with an anion exchange membrane adsorber serving as a depth filter mimic to purify the load material in preparation for the subsequent IEX operations.^3^, ^4^ This established a fully automated industry‐standard micro‐scale platform encompassing all typical major unit operations within the purification sequence (Figure 1). These studies also demonstrated equivalence in process performance between scales while highlighting the potential of HTPD for accelerated process development. However, this integration remained incomplete due to one key technological limitation: the lack of a micro‐scale, automated method for accurate pH measurement and control.

Low pH VI is a critical viral safety step that ensures the inactivation of enveloped viruses present in the product stream following protein A capture.^7^ The operation typically involves lowering the process intermediate to a pH ≤3.6^8^ for a defined hold period before neutralization to pH 5.5 to restore protein stability. Precise pH control during both acidification and neutralization is essential, as deviations can result in incomplete inactivation or induce aggregation of the antibody product.^9^, ^10^ While pH measurement is routine at the laboratory scale, existing micro‐scale systems lack integrated probes capable of detecting within the required acidic range while maintaining compatibility with automated liquid‐handling platforms. Consequently, pH must be measured off deck using manual methods, interrupting automation and limiting the integration of the VI step with preceding and subsequent operations, including Sartobind® Q and IEX chromatography.

Research has focused on developing and implementing automated pH measurement approaches for micro‐scale or high throughput applications, yet these methods remain unsuitable for integration within automated low pH viral inactivation workflows. Spectrophotometric dye‐based assays, such as the absorbance‐based method described by BMG LABTECH, enable rapid, parallel pH determination in microplate formats.^11^ However, these assays are typically optimized for near‐neutral pH ranges (approximately pH 6–8) and rely on the addition of pH‐sensitive dyes to the sample. The introduction of such dyes must be accounted for within the purification process, as they could interfere with subsequent analytical or chromatographic steps. Furthermore, the optical response of these assays is highly sensitive to turbidity, protein concentration, and buffer composition, which compromises accuracy under the strongly acidic conditions required for viral inactivation. Furthermore, automated multi‐probe systems, such as the Unchained Labs Big Kahuna and Junior platforms, employ motorized probe arms to perform high throughput pH measurements across microplates and have proven effective for formulation screening and stability studies.^12^ However, these systems rely on direct probe contact, require washing and equilibration between measurements, and are not designed to operate within the low pH range or small working volumes typical of micro‐scale bioprocessing. Moreover, their closed hardware configuration prevents seamless integration with general‐purpose liquid‐handling systems such as the Tecan Freedom EVO®, limiting their suitability for fully automated process workflows. More recently, optical and microfluidic sensor‐based approaches, such as those employing fluorescence or absorbance lifetime detection, have been proposed for high‐throughput pH measurement.^13^ While these demonstrate promising precision and miniaturization, they remain prototype systems that are not commercially available and therefore cannot be readily implemented within an industrial automation platform.

More recently, Murray et al. (2023), demonstrated the integration of Mettler Toledo InLab® Micro pH probes directly onto a Tecan EVO 200 liquid handling platform, coupled with a feedback control loop for automated protein solution titration to a low pH target and subsequent neutralization.^14^ This work represents an important advancement in automating solution adjustment within chromatography development workflows and clearly establishes the feasibility of closed‐loop pH control on a liquid‐handling deck. However, the approach relies on dedicated integrated probe hardware, external pH meters, and custom system configuration, which may limit accessibility and scalability across laboratories lacking specialized instrumentation.

Despite advances in automated pH analysis, no existing technique provides the required combination of low‐pH sensitivity, small‐volume compatibility, and direct integration with automated liquid‐handling platforms. This highlights the need for an automated, on‐deck pH measurement and control method capable of operating across the acidic range relevant to viral inactivation, which this study seeks to address.

To overcome this limitation, this work presents the development and integration of an on‐deck pH measurement method within a micro‐scale HTPD platform designed for mAb downstream purification. In contrast to probe‐based integration approaches, the method employs flat‐bottom 96‐well microplates equipped with pH‐responsive optical sensors immobilized onto the base of each well. These sensors are fully compatible with automated liquid‐handling environments and standard plate readers, enabling non‐invasive, real‐time detection of pH across the range required for viral inactivation using off‐the‐shelf, low‐cost, multipurpose laboratory hardware. By demonstrating that pH control functionality can be achieved without dedicated integrated probe systems, this work extends prior advances and provides a practical, scalable solution for broader implementation of automated low pH processing workflows.

Optical pH sensors of this type have previously been used for automated at‐line pH measurement in milliliter‐scale bioreactors for high throughput bioprocess design.^15^ In those applications, pH was determined non‐invasively by integrating pH‐sensitive sensor spots directly into the base of microtiter wells and employing fluorescence lifetime detection using a dual‐lifetime referencing method for enhanced accuracy.^16^ In this approach, the sensor dye is excited by light and emits fluorescence whose decay time changes as a function of pH. Measuring this decay time, rather than fluorescence intensity, makes the signal less sensitive to variations in dye concentration, light intensity, or optical path length. Dual‐lifetime referencing further improves robustness by incorporating a second, pH‐insensitive reference dye; the reference signal is used to internally normalize the pH‐sensitive signal, compensating for optical disturbances such as turbidity or minor positioning differences. This approach enabled continuous, automated monitoring and feedback control of pH during cultivation. While such sensors have traditionally been applied to upstream operations, their repurposing here for downstream processes offers a novel solution for precise, small‐volume pH measurement in automated viral inactivation workflows.

This functionality was further enhanced through the implementation of a feedback control strategy, which enabled autonomous adjustment of acid and base additions to achieve specific target pH values during both the acidification and neutralization phases. To demonstrate the capability of the fully integrated micro‐scale platform to support downstream process development, the system was subsequently applied to assess the impact of buffer pH on CEX chromatography performance. This assessment highlighted the platform's ability to evaluate critical process parameters within an identical automated workflow, reinforcing its value as a rapid screening and optimization tool for polishing operations.

By incorporating automated pH measurement and control, this work represents a key advancement toward fully integrated micro‐scale downstream process development. The system establishes a platform for data‐driven pH adjustment, facilitating reproducible execution of low pH VI while enabling direct progression to subsequent operations such as membrane adsorber or ion exchange purification. Moreover, the approach enhances process robustness, reduces manual intervention, and extends the applicability of HTPD to non‐chromatographic unit operations. Ultimately, the on‐deck pH measurement method and integrated feedback control architecture advance the vision of a fully automated, end‐to‐end HTPD workflow for mAb purification.

## 2 MATERIALS AND METHODS

### 2.1 Material source

The material source is a clarified harvest of a fully human IgG1κ mAb produced in cultures of CHO cells.

### 2.2 Micro‐scale unit operations

#### 2.2.1 Protein a chromatography

This operation utilized up to eight OPUS® RoboColumns® (Repligen, United States), operated in parallel, pre‐packed with MabSelect SuRe resin (Cytiva, United States). The column parameters are presented in Table 1.

**TABLE 1:** OPUS® RoboColumn® MabSelect SuRe column parameters used for the micro‐scale protein A chromatography operation.

Column	Inner diameter	Gel bed height	CV	mAb Titer	Load density	Residence time
	(cm)	(cm)	(mL)	(mg/mL)	(mg/mL)	(min)
MabSelect SuRe	0.50	3.00	0.59	1.06	25	4.50

The starting material for this operation was 13.9 mL of clarified harvest per column (1.06 g/L protein concentration), which was thawed in a water bath at 25°C, before being placed, along with the buffers, onto the deck of the Tecan Freedom EVO® 200 (Tecan, Switzerland).

Once the consumables were defined in the Robotools (v1.11.2) script and matched to the deck layout created in Freedom EVOware® (v2.8 SP7) (Tecan, Switzerland), the workflow was designed and created, which was based upon a laboratory‐scale protocol and following manufacturer's recommendations for operation on the resin. The workflow was then exported as a series of commands to Freedom EVOware® (Tecan, Switzerland). The implementation of the micro‐scale chromatographic operations closely mimics that of the laboratory‐scale operation across the protocol steps, including (1) equilibration; (2) load; (3) post‐load wash; (4) wash; (5) elution; (6) regeneration; (7) sanitisation; (8) re‐equilibration, and; (9) storage. In the micro‐scale format, where in‐line UV detection was not available, pooling was instead standardized to a fixed fraction of the eluate, typically corresponding to 4 CV.

#### 2.2.2 Low pH viral inactivation

The low pH VI operation was designed and constructed using Freedom EVOware (v2.8 SP7) (Tecan, Switzerland), the protocol of which was based upon the existing laboratory‐scale operation. The workflow was then executed, in sequence with the preceding and proceeding operations, on the Tecan Freedom EVO® 200 (Tecan, Switzerland).

The actions performed by the robotic station involved diluting the pooled protein A eluate 1:4 with the protein A elution buffer (50 mM acetate, pH 3.8) to a target conductivity of ≤0.5 mS/cm, prior to the low pH hold. This dilution was implemented to minimize aggregation, reflecting the aggregation‐prone nature of the mAb under acidic conditions.^3^ The volume of acidification solution (3 M acetic acid) added to the diluted protein A eluate was adjusted depending on the desired pH to be achieved for low pH hold. For example, ~ 10%–12% (v/v) of start material amount was added to achieve a pH of 3.5 through acidification solution, and ~14%–15% (v/v) of start material amount was added to achieve a pH of 5.5 through neutralization solution (2 M tris). Following acidification, the eluate was held for 45 min, before addition of neutralization solution. For comparison, reference pH values of the samples were measured off‐deck using the pH electrode InLab® Micro (Mettler Toledo, United States) following acidification and neutralization.

#### 2.2.3 Sartobind® Q workflow design and task execution

As demonstrated in our previous work, this unit operation was integrated within the micro‐scale platform to serve as a surrogate for depth filtration.^4^ The micro‐scale Sartobind® Q operation was designed and constructed using Robotools (v1.11.2), the protocol of which was developed specifically for the integrated micro‐scale platform. Once successfully designed and tested, the workflows were then executed on the Tecan Freedom EVO® 200 (Tecan, Switzerland). The starting material for this operation was 0.5 mL of the neutralized protein A eluate per well, which was placed, along with the pre‐prepared buffers, onto the deck of the Tecan Freedom EVO® 200 (Tecan, Switzerland).

The actions of the robotic liquid handler involve (1) equilibration; (2) load; (3) adjustment of the centrifuge balance plate; (4) centrifugation; (5) shaking and collection of flowthrough; (5) wash; (7) adjustment of the centrifuge balance plate; (8) centrifugation; and (9) shaking and collection of wash.

#### 2.2.4 Cation exchange chromatography

This operation utilized up to eight OPUS® RoboColumns® (Repligen, United States), operated in parallel, pre‐packed with commercially available CEX resin (Cytiva, United States). The column parameters are presented in Table 2.

**TABLE 2:** OPUS® RoboColumn® Capto™ SP ImpRes column parameters utilized for the micro‐scale cation exchange chromatography operation.

Column	Inner diameter	Gel bed height	CV	mAb titer	Load density	Residence time
	(cm)	(cm)	(mL)	(mg/mL)	(mg/mL)	(min)
Capto™ SP ImpRes	0.50	3.00	0.59	5	25	4

The starting material for this operation was 20 mL of the Sartobind® Q flowthrough per column, which was placed, along with the pre‐prepared buffers, onto the deck of the Tecan Freedom EVO® 200 (Tecan, Switzerland).

Once the consumables were defined in the Robotools (v1.11.2) script and matched to the deck layout created in Freedom EVOware® (Tecan, Switzerland), the workflow was designed and created, which was based upon a laboratory‐scale protocol and following the manufacturer's recommendations for operation of the resin. The workflow was then exported as a series of commands to Freedom EVOware® (Tecan, Switzerland). The actions performed by the robotic liquid handler include (1) equilibration; (2) load; (3) wash; (4) gradient elution; (5) regeneration; (6) re‐equilibration; (7) neutralization; and (8) storage. Elution was performed using a gradient of increasing conductivity. As automated liquid handlers operate in a discrete rather than continuous manner, a pseudo‐linear gradient was implemented by increasing the NaCl concentration from 15 to 150 mM across 12 incremental steps. The gradient profile was pre‐calculated based on the total number of steps, and the corresponding buffers were prepared in advance and positioned on the deck prior to operation.

### 2.3 Automation of pH measurement

To enable the integration of low pH VI within the automated micro‐scale platform, an on‐deck pH measurement system was established using optical sensor technology. Flat‐bottom 96‐well microplates were immobilized with pH‐responsive sensor spots, LV1R‐1951‐01_2 (PreSens GmbH, Germany). Each sensor consisted of an indicator dye, whose fluorescence is pH‐dependent, and a reference dye, unaffected by pH. Fluorescence intensity ratios between the indicator (*I*_*indicator*_) and reference (*I*_*reference*_) signals were measured using a Spark® Multimode Microplate Reader (Tecan, Switzerland) with a monochromator bandwidth of 20 nm. Excitation and emission settings are summarized in Table 3.

**TABLE 3:** Excitation and emission filter pairs to detect the *I*_indicator_ and *I*_reference_ using the Spark® Multimode Microplate Reader with a monochromator bandwidth of 20 nm.

	Excitation filter	Emission filter	Detected fluorescence
Filter pair 1	485 nm	538 nm	*I*_indicator_
Filter pair 2	485 nm	620 nm	*I*_reference_

The fluorescence ratio, *I*_*R*_, was then calculated and correlated to the pH of the sample as shown:

(1) $$I_{R}=\frac{I_{\text{indicator}}}{I_{\text{reference}}}$$

A 12‐point calibration, covering the pH range 3–8, was conducted to determine four constants: *I*_min_ (the minimum sensor signal at low pH), *I*_max_ (the maximum signal at high pH), *dpH* (the slope factor describing the steepness of the response curve), and *pH 0* (the midpoint pH at which the sensor response is half of its maximum). The calibration curve was plotted (Figure S1), and constants were calculated. Prior to measurement, the calibration buffers were incubated in the microplate for 45 min at 27°C to equilibrate the sensor spots.

The measured pH was then calculated using the following equation, which requires the four calibration constants:

(2) $$\mathrm{pH}=\frac{\left(I_{\min}-I_{\max}\right)}{1+\mathrm{Exp}\left(x-\frac{\mathrm{pH}0}{\mathrm{dpH}}\right)}$$

where *x* corresponds to the measured *I*_*R*_ value.

For experimental runs, sacrificial sample volumes of 200 μL were transferred to the sensor‐containing wells. To ensure comparability between calibration and measurement conditions, 10 μL of 1 M NaCl was added directly to the wells to adjust ionic strength. This approach minimized aggregation risks by avoiding salt addition to the pooled sample material. Plates were incubated for 45 min at 27°C prior to measurement.

For conductivity screening experiments, samples were prepared using a defined dilution and salt addition protocol to evaluate the effect of ionic strength on pH measurement accuracy. 200 μL of protein A eluate process intermediate was transferred into a 96‐well deep‐well plate and diluted 1:4 with protein A elution buffer. Subsequently, defined volumes of 1 M NaCl (0, 20, or 40 μL) were added to the diluted samples to adjust ionic strength. The solutions were mixed using aspirate‐dispense cycles to ensure homogeneity prior to measurement. An aliquot of each prepared sample was then transferred to the sensor‐containing microplate wells for pH measurement. The microplate was incubated at 27°C for 45 min prior to measurement. Importantly, measurement accuracy is ensured by operating within the validated calibration range of the sensor and maintaining consistent ionic strength between calibration and measurement conditions.

For comparability studies, when pH measurement was performed off‐deck, samples were manually withdrawn from the micro‐scale platform and analyzed using a pH Sensor InLab® Micro electrode (Mettler Toledo, United States). Each sample was measured immediately after collection to minimize pH drift, and the recorded value was used to determine subsequent buffer additions before resuming automated on‐deck processing.

#### 2.3.1 Feedback control strategy

Following validation of the measurement workflow, a feedback control strategy was implemented to automate pH adjustment during low pH VI. The system was built using Momentum™ scheduling software (Thermo Fisher Scientific, United States) in combination with custom Python scripts, enabling coordination between the liquid handler, robotic transport, and plate reader. After each process step requiring pH measurement, the liquid handler dispensed processed material into microplate wells containing defined volumes of acid or base buffer together with 40 μL of 1 M NaCl, the latter included only in the microplate wells and not in the pooled bulk sample to prevent aggregation that could interfere with downstream purification. The number of wells used was adaptable, allowing flexibility in screening different buffer conditions. The prepared microplate was then transferred by the Spinnaker robotic arm to the Spark® reader (Tecan, Switzerland), where pH was measured via phase‐shift calculations using pre‐determined calibration constants. The resulting data were parsed by a Python‐based control script, which calculated the buffer‐to‐sample ratio required to reach the target pH. A worklist was then automatically generated and executed by the liquid handler to apply the ratio that was closest to the target pH from the ones that were created and measured to the pooled bulk material. This workflow was performed sequentially for both the acidification and neutralization steps of low pH VI.

### 2.4 Mixing studies

Mixing efficiency at the micro‐scale was assessed using a colourimetric dye assay with bromophenol blue, designed to evaluate the mixing strategy employed on the Tecan Freedom EVO® 200 platform (Tecan, Switzerland). This strategy consisted of three aspirate–dispense cycles applied to the target solution.

Bromophenol blue was selected as a pH‐sensitive indicator due to its distinct color transition from yellow at pH <3 to purple at pH >4.6. Following buffer addition to adjust the solution pH, mixing was performed according to the mixing strategy. Absorbance at 590 nm was recorded using a microplate reader 60 s after mixing initiation. To ensure representative sampling, aliquots were collected from three discrete locations within the well and averaged.

Mixing homogeneity was quantified using the dimensionless parameter *M*_*60*_:

(3) $$M_{60}=\frac{A590_{60s}}{A590_{\infty }}$$

where A590,60s represents the absorbance measured 60 s after mixing, and A590,∞ corresponds to the absorbance of a fully mixed reference. This assay provided a basis for evaluating the suitability of existing mixing strategies for application to the low pH VI step.

#### 2.4.1 Design and integration of a custom trough carrier

To enable seamless integration of the low‐pH viral inactivation (VI) step within the automated micro‐scale purification sequence, a custom 200 mL trough carrier was designed and fabricated for use on the Bioshake module of the Tecan Freedom EVO® 200 platform. The carrier was constructed with an SBS‐standard footprint (128 × 85 mm) to ensure full compatibility with the existing deck layout and robotic workflows. To minimize dead volume during automated aspirate‐dispense cycles, the trough was angled at 5° relative to the horizontal plane, enabling efficient liquid handling and reproducible mixing under micro‐scale conditions.

The carrier was modeled in FreeCAD (v1.0.0), sliced using PrusaSlicer (v2.7.3; Prusa Research, Czech Republic), and 3D printed on an Original Prusa MK4 system (Prusa Research, Czech Republic) using Prusament PETG filament. PETG was selected for its mechanical stability, chemical resistance, and durability during repeated automated operations. The dimensional accuracy of the final print was verified to ensure a secure fit on the Bioshake module and precise alignment with the liquid handling system.

During operation, the 200 mL trough contained the diluted, pooled protein A eluate immediately prior to the low pH VI step. Acidification and neutralization buffers were dispensed under automated control, with buffer‐to‐sample ratios defined by prior screening studies. Mounted on the Bioshake module, the trough supported controlled mixing, adjustable in both duration and rpm, to ensure homogeneous buffer distribution.

**FIGURE 2:**
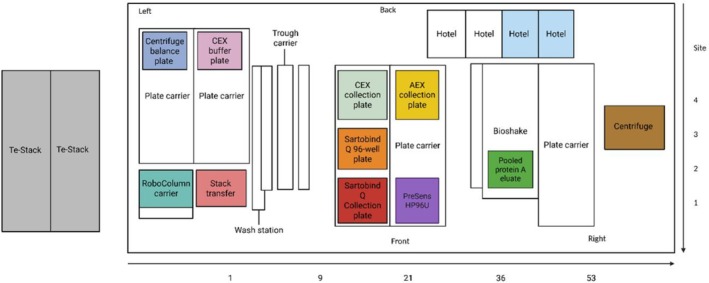
Deck configuration of the Tecan Freedom EVO® 200 platform for the integrated mAb purification sequence. This configuration supports the automated execution of key downstream processing steps, including protein A chromatography, low pH viral inactivation, Sartobind® Q membrane chromatography, CEX, and AEX polishing. The x‐axis denotes the horizontal position of labware and carriers on the deck, while the y‐axis indicates site numbers used by the Liquid Handling Arm and Robotic Manipulator Arms for coordinated navigation and operation. The 200 mL trough used for material distribution is positioned on a custom‐designed trough carrier (green), and the pH measurement plate (PreSens HP96U) is shown in purple. This layout represents the standard setup for a typical mAb purification sequence, enabling seamless integration of liquid handling, pH measurement, and column‐ and plate‐based purification steps. Figure created with BioRender.com .

This configuration enabled reproducible pH adjustment during both acidification and neutralization, ensuring consistent execution of the low pH VI step. The integration of the custom trough carrier provided an automation‐compatible solution for handling larger micro‐scale volumes, maintaining reliable mixing while minimizing aggregation risk. As shown in Figure 2, the custom‐designed carrier (green) served as the designated position for the pooled protein A eluate within the deck layout, supporting full integration of all downstream unit operations within the automated mAb purification sequence.

### 2.5 Analytical testing

#### 2.5.1 Protein concentration

MAb concentration was determined using the Infinite M200 Pro plater reader (Tecan, Switzerland). The collected fractions from the unit operations were diluted by a factor of 100 in elution buffer before being transferred to a UV‐Star® 96 well plate (Greiner Bio‐One, Germany). These were then measured at A280, A320, A900, and A977, which accounts for pathlength correction:

(4) $$\text{Pathlength}\;\left(\mathrm{cm}\right)=\frac{A_{977}-A_{900}}{K-\text{Factor}}*10\;\mathrm{mm}$$

where 0.173 was assumed for the *K‐Factor* based upon the elution buffer used. The corrected absorbance is given by:

(5) $$A_{\text{corrected}}=\frac{A_{\mathrm{raw}}*K-\text{Factor}}{A_{977}-A_{900}}*10\;\mathrm{mm}$$

Mass concentration is then determined by:

(6) $$\text{Mass concentration}\;\left(\frac{\mathrm{mg}}{\mathrm{mL}}\right)=\frac{A_{\text{corrected}}}{\epsilon *d}$$

where *A* is absorbance, *ϵ* is extinction coefficient (molar adsorption coefficient), and *d* is pathlength (cm).

The protein concentration of process intermediates generated within the integrated micro‐scale platform was determined using the Lunatic microfluidic UV/Vis spectrophotometer (Unchained Labs, United States). This high throughput system employs fixed‐pathlength microfluidic cuvettes, allowing precise absorbance measurements across a dynamic range of 0.03–275 mg/mL without the need for manual dilution.

For each measurement, 2 μL of sample was loaded into the microfluidic plate. Full absorbance spectra were recorded over the range 230–350 nm, and protein concentration was calculated from the absorbance at 280 nm using the Beer–Lambert law with an E1% of 15.1.

#### 2.5.2 High molecular weight component quantification

High molecular weight components (HMWC) were quantified by SE‐HPLC. Analyses were carried out on a Vanquish HPLC system (Thermo Fisher Scientific, United States) equipped with a silica‐based SEC column (Acquity BEH200, 1.7 μm, 4.6 × 150 mm, Waters, United States). Samples were resolved under isocratic elution conditions in accordance with the manufacturer's recommendations for column operation. The mobile phase consisted of 100 mM Bis‐Tris propane, 220 mM NaCl, pH 7, which was diluted 5‐fold prior to use. The flow rate was set to 0.3 mL/min, and the total run time was 12 min. The column was maintained at 35°C, and the autosampler was held at 6°C. Samples were injected using a 25 μL sample loop, with an injection volume range of 0.2–25 μL based on the protein concentration. A target column load of 20 μg total protein was used per injection. UV detection was performed at 280 nm with a 5 Hz data collection rate, 1‐s response time, and a peak width setting of 0.1 min using a 2.5 μL flow cell.

### 2.6 Statistical analyses

#### 2.6.1 Paired *t*‐test

A paired *t*‐test was applied when comparing two related groups, such as matched data collected from the same sample under two different conditions or time points. This test determines whether the mean of the differences between paired observations significantly differs from zero. The null hypothesis (*H*₀) stated that there was no significant difference between the paired groups, while the alternative hypothesis (*H*₁) indicated that a statistically significant difference existed. The test statistic was calculated as:

(7) $$t=\frac{\bar{d}}{\frac{s_{d}}{\sqrt{n}}}$$

where $\bar{d}$ denotes the mean of the differences between paired observations, $s_{d}$ represents the standard deviation of these differences, and n is the number of paired samples. The numerator reflects the average difference between pairs, and the denominator is the standard error of the mean difference. The test statistic, *t*, was compared against a *t*‐distribution with n−1 degrees of freedom. A two‐tailed *p*‐value was calculated for each comparison, using a significance threshold of *α* = 0.05. *p*‐Values less than 0.05 were considered sufficient to reject the null hypothesis, implying that the observed difference was unlikely to be due to random chance.

## 3 RESULTS AND DISCUSSION

### 3.1 Development of the integrated pH measurement method

The pH measurement method was initially implemented during the low pH VI operation in isolation. Accurate monitoring of the pH during both the hold and neutralization phases is critical to assess mAb stability by minimizing the risk of aggregate formation.^9^ To evaluate the system, three key process intermediates were selected: diluted protein A eluate, acidified protein A eluate, and neutralized protein A eluate.

For the conductivity screening experiments presented in Table 4, samples were prepared using a defined dilution and salt addition protocol to evaluate the effect of ionic strength on pH measurement accuracy. Specifically, 200 μL of protein A eluate process intermediate was first transferred into a 96‐well deep‐well plate and diluted 1:4 with protein A elution buffer. Subsequently, defined volumes of 1 M NaCl (0, 20, or 40 μL) were added to the diluted samples to adjust the ionic strength. The solutions were mixed using aspirate‐dispense cycles to ensure homogeneity prior to measurement. An aliquot of each prepared sample was then transferred to the sensor‐containing microplate wells for pH measurement. To ensure consistency between calibration and measurement conditions, the microplate was incubated at 27°C for 45 min prior to measurement using the Spark® Multimode Microplate Reader. The pH values were calculated using previously established calibration constants. Reference pH values were independently determined using a pH Sensor InLab® Micro electrode, and deviations between reference and measured pH values are summarized in Table 4. This preparation method was used exclusively for the conductivity screening study to enable controlled and reproducible evaluation of sensor performance across defined ionic strength conditions.

**TABLE 4:** Deviations between the expected and measured pH values for protein A, acidified protein A, and neutralized protein A samples measured at conductivities of 0.2/0.4, 3, and 6 mS/cm.

Sample	Conductivity (mS/cm)	Expected pH	Reference pH	Deviation (%)
0 μL 1 M NaCl				
Protein A Eluate	0.2	3.8	4.5 ± 0.006	18.4
Acidified protein A Eluate	0.2	3.5	4.4 ± 0.002	25.7
Neutralized protein A Eluate	0.4	5.5	5.2 ± 0.017	−5.5
20 μL 1 M NaCl				
Protein A Eluate	3	3.8	4.1 ± 0.004	7.9
Acidified protein A Eluate	3	3.5	3.9 ± 0.002	11.4
Neutralized protein A Eluate	3	5.5	5.8 ± 0.010	5.5
40 μL 1 M NaCl				
Protein A Eluate	6	3.8	3.8 ± 0.002	−0.7
Acidified protein A Eluate	6	3.5	3.5 ± 0.010	−0.6
Neutralized protein A Eluate	6	5.5	5.4 ± 0.007	−1.8

*Note*: Errors represent the standard deviation from triplicate measurements.

Table 4 summarizes the deviations between reference and measured pH values across the tested conductivity range. Initial measurements performed without NaCl addition showed significant deviations (*p* < 0.05) between the optical sensor and reference electrode measurements. Upon addition of 20 μL of 1 M NaCl, the sample conductivity increased and measurement deviations were reduced, although statistically significant differences remained. This progressive improvement in agreement with increasing conductivity indicates that sensor response was influenced by solution ionic strength. This behavior is consistent with the operating principle of optical pH sensors, which rely on the protonation‐deprotonation equilibrium of an immobilized indicator dye governed by proton activity. While pH is formally defined in terms of proton activity, optical sensors are typically calibrated under defined ionic strength conditions and do not explicitly compensate for changes in activity coefficients. Proton activity is related to proton concentration through an ionic‐strength‐dependent activity coefficient, therefore, variations in ionic strength can alter the relationship between concentration and activity relative to calibration conditions. At low ionic strength, these effects become more pronounced, which can lead to discrepancies between optical sensor measurements and reference electrode measurements.

Increasing the NaCl volume to 40 μL, corresponding to a conductivity of 6 mS/cm, further improved agreement between reference and measured values. Under these conditions, deviations became statistically non‐significant (*p* > 0.05) across all tested samples, confirming that increased ionic strength stabilized the sensor response. Importantly, no increase in aggregate formation attributable to NaCl addition was observed, accentuating that the ionic strength adjustment required for accurate pH measurement did not adversely affect protein stability under the conditions evaluated. These findings demonstrate that controlling sample conductivity is essential for accurate on‐deck pH measurement and validate the robustness of the optimized measurement protocol within the targeted process‐relevant pH range.

In contrast, during automated process runs and feedback‐controlled pH adjustment, sample preparation was performed directly within the sensor‐containing microplate wells as part of the integrated automated workflow, without intermediate dilution in a deep‐well plate. A sacrificial volume of 200 μL of process material was transferred directly into wells containing immobilized optical pH sensors, along with predefined volumes of acid or base buffer and 10 μL of 1 M NaCl to ensure consistent ionic strength during measurement. The transferred volume was used solely for pH determination and was not returned to the pooled process material. Importantly, NaCl was added only within the measurement wells and not to the bulk process intermediate, thereby preserving the original process conditions and avoiding increased aggregation risk. Following preparation, the microplate was incubated at 27°C for 45 min to allow equilibration between the sample and optical sensor spots. The plate was then transferred to the Spark® Multimode Microplate Reader, and pH values were calculated using the established calibration constants. This approach enabled rapid, automated, and reproducible pH measurement fully integrated within the robotic workflow.

### 3.2 Development of the pH measurement feedback control strategy

With the successful development of an automated pH measurement method, a feedback control strategy was implemented as a core component of the low pH VI operation. This integration enables the fully autonomous execution of both acidification and neutralization steps, eliminating the need for manual intervention and significantly improving workflow efficiency. More importantly, this automation allows for precise determination of acid and base buffer volumes required to achieve specific target pH values, thereby enhancing the robustness and reproducibility of the method.

The feedback control strategy establishes the micro‐scale platform as an effective assessment tool for different mAb molecules. By determining the required volumes of pH adjustment buffers for each molecule, optimal conditions can be rapidly identified and applied to bulk pooled material. This standardized approach reduces manual titration efforts, minimizes operator variability, and accelerates development timelines while ensuring comparability across molecules and batches.

The pH feedback strategy was constructed using Momentum scheduling software in conjunction with internally developed Python control scripts. Following each process step requiring pH measurement, the liquid handler prepared test samples by dispensing the processed material into microplate wells containing 1 M NaCl and varying volumes of acid or base buffer. Notably, 1 M NaCl was added only to the microplate wells and not to the pooled sample material, in order to avoid inducing aggregation, which could otherwise interfere with subsequent purification steps and would require additional mitigation strategies.

The number of wells used was adaptable, allowing the platform to accommodate various screening designs depending on the number of buffer conditions tested. The prepared microplate was transported by the Spinnaker robotic arm to a microplate reader, which measured pH via phase‐shift calculations based on pre‐calibrated constants. The resulting data were parsed by a Python‐based server, which identified the buffer‐to‐sample ratio that most closely aligned with the target pH. A worklist was then generated for the liquid handler to apply the ratio that was closest to the target pH from the ones that were created and measured, to the pooled bulk material. This procedure was performed twice for each sample: once for the acidification step and once for neutralization.

**FIGURE 3:**
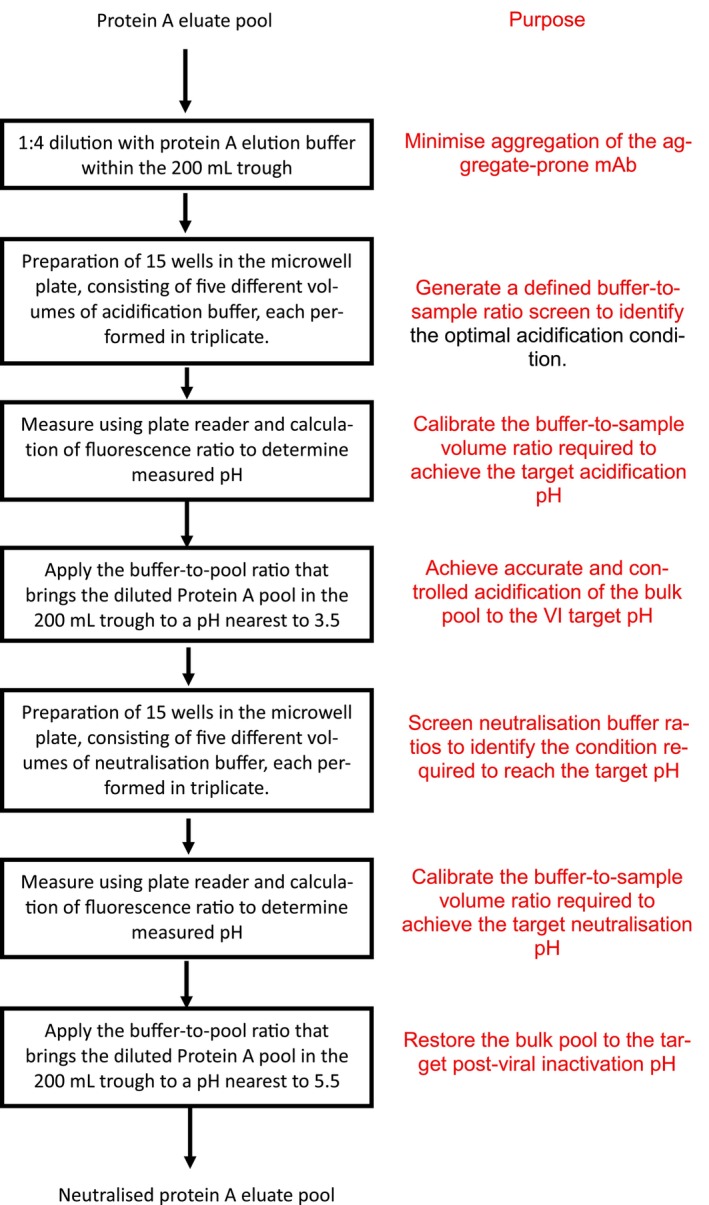
Process flowsheet illustrating the integration of on‐deck pH measurement and a feedback strategy within the low pH viral inactivation operation. The diagram outlines the automated workflow from protein A eluate pooling and controlled dilution, through buffer‐to‐sample ratio screening for acidification, calibration of the required buffer volume, and application of the optimized ratio to achieve the viral inactivation target pH. The sequence is subsequently repeated for neutralization, including screening of neutralization buffer ratios, calibration based on measured pH response, and application of the selected ratio to restore the pool to the post‐viral inactivation target pH. Each step is annotated with its operational purpose, emphasizing the role of the feedback strategy in calibrating buffer additions, ensuring accurate pH adjustment, and maintaining reproducible process conditions across samples.

Figure 3 illustrates the fully automated feedback control strategy seamlessly integrated into the low pH VI operation. The combination of automation, parallelization, and data‐driven optimization enables rapid, hands‐free execution of this critical process step. As a result, the system functions as an efficient and scalable screening tool for mAb process development, facilitating early identification of molecule‐specific pH behavior while maintaining throughput, reproducibility, and minimal resource consumption.

The feedback control strategy provides clear advantages over conventional off‐deck manual pH adjustment. The manual approach, previously developed and implemented in micro‐scale low pH VI studies, relies on microprobe‐based titration involving repeated sampling, reagent addition, and pH measurement using an external benchtop pH meter.^3^ These steps introduce variability, increase sample handling, elevate contamination risk, and significantly extend processing time. In contrast, the automated feedback strategy achieves target pH values directly on deck in a fully autonomous manner.

**FIGURE 4:**
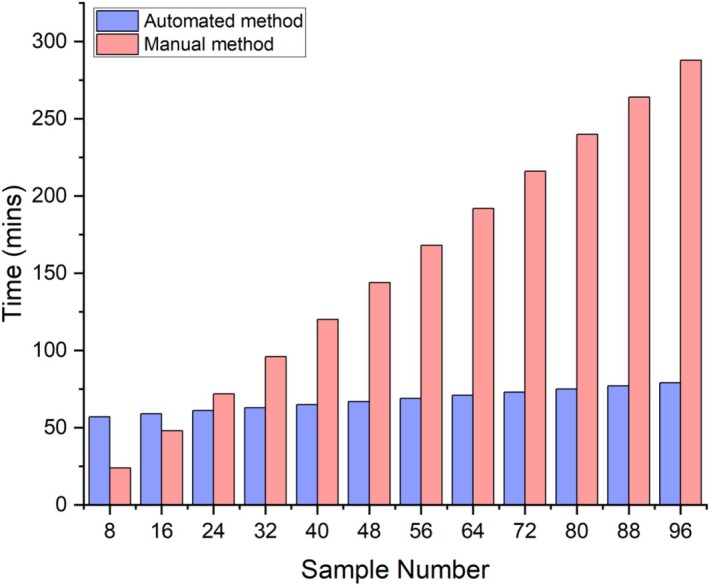
Measurement time (minutes) using the automated on‐deck feedback control method (blue bars) and conventional manual off‐deck adjustment (red bars) for increasing sample numbers. Automated processing time includes a fixed 45‐min incubation period for plate reader equilibration and analysis, followed by liquid handling operations, with approximately two additional minutes required per block of eight samples. Manual processing time was calculated assuming sequential handling and measurement of each sample using a pH Sensor InLab® Micro electrode, with approximately 1 min required per individual measurement. The automated method therefore exhibits stepwise scaling behavior, whereas the manual method increases linearly with sample number.

Beyond improved accuracy, the platform also delivers substantial operational advantages. Figure 4 highlights the scalability of the automated system, demonstrating a fundamentally different time‐sample number relationship compared to manual pH measurement. While manual processing time increases almost linearly with sample number, the automated workflow exhibits stepwise scaling behavior. At low sample numbers, total processing time is influenced by the fixed 45‐min incubation required during plate reader analysis. This incubation represents an inherent analytical requirement and explains the comparatively higher time demand at very small sample numbers. However, once incubation is complete, the system leverages the parallelization capabilities of the liquid handling platform. Buffer addition, mixing, plate handling, and data processing are executed in parallel, and each additional block of eight samples requires only approximately two additional minutes of processing time. Consequently, total processing time increases in small increments per eight samples rather than proportionally per individual well.

In contrast, manual pH measurement using a microprobe requires sequential handling and measurement of each well, taking approximately 1 min per sample. Every condition must be manually, measured, recorded, and adjusted, resulting in a direct, linear relationship between time and sample number. This not only increases labor intensity as throughput rises but also requires continuous operator presence. The automated workflow, by comparison, is entirely walk‐away. Once initiated, the platform performs buffer addition, mixing, pH measurement, and ratio selection autonomously without further user intervention. This decoupling of operator time from sample number represents the most significant operational advantage of the system, enabling unattended processing of large experimental matrices.

The walk‐away capability fundamentally expands the accessible experimental space. Multiple input materials, parallel protein A operations, or different process intermediates can be evaluated simultaneously within a single automated run. For example, as shown in Figure 3, the demonstration workflow utilized 15 wells within a 96‐well microplate; however, the format readily supports expansion to the full 96‐well capacity within a single plate, substantially increasing throughput without additional manual effort. Replicates can be incorporated without increasing operator burden, allowing detection of small but meaningful differences in pH response or process sensitivity. Furthermore, the system can function as a high throughput screening tool, enabling systematic evaluation of stabilizers, buffer compositions, titration strategies, or molecule‐specific behaviors under defined conditions. Rather than limiting experimentation to a small number of manually manageable conditions, the automated platform allows broad parameter screening within realistic timeframes.

The implementation requires minimal additional capital investment, as it leverages standard laboratory infrastructure such as plate readers and liquid handling platforms that are already available in most development laboratories. The system successfully meets all technical performance requirements, as evidenced by precise pH convergence, reproducibility, and automation reliability across both acidification and neutralization phases. By integrating pH sensing, data processing, and buffer addition into a continuous control cycle, the system eliminates manual intervention while maintaining consistent measurement conditions across all samples. This standardization reduces experimental noise, enhances reproducibility, and enables precise determination of buffer volumes required to reach target pH values, minimizing reagent use and avoiding overshoot, a common limitation of manual titration. These improvements are particularly valuable at micro‐scale, where small volumetric deviations can cause disproportionate pH shifts.

Overall, the on‐deck feedback control strategy accelerates the adjustment process, improves accuracy, and standardizes pH control in both acidification and neutralization phases. Most importantly, its fully automated, walk‐away operation transforms pH adjustment from a labor‐intensive bottleneck into a scalable, high throughput screening capability. By providing traceable, data‐driven operation aligned with large‐scale process design principles, the system enhances robustness, reproducibility, and experimental flexibility, establishing it as a scalable and superior alternative to manual pH adjustment for downstream micro‐scale VI and polishing workflows.

### 3.3 Integration within the automated micro‐scale platform

To integrate the low pH VI step, coupled with pH measurement feedback control, with the subsequent Sartobind® Q operation, a robust and appropriately sized mixing vessel is required. This vessel must accommodate a 1:4 dilution of the pooled eluate with elution buffer and enable efficient mixing. Such mixing is critical to ensure seamless automation and integration of the low pH VI step with both the preceding protein A chromatography and downstream purification processes. Using a one‐size‐fits‐all approach to mixing may compromise product quality. For instance, the previously employed mixing strategy, which comprises three aspirate‐dispense cycles, applied to this significantly larger volume of diluted eluate, yielded *M*_60_ = 0.63 ± 0.18 (*n* = 3). *M*_60_ is a dimensionless parameter used to quantify mixing efficiency within a defined time period, typically 60 s after mixing initiation. It is calculated as the ratio of the measured absorbance at 60 s to that of a fully mixed reference, providing a rapid and comparative measure of how effectively solutions are homogenized. This reduction in mixing efficiency underscores the limitations of the existing method and the potential impact on product consistency and quality.

To address this, a custom 200 mL trough carrier was designed and 3D‐printed for use on the Bioshake module of the Tecan Freedom EVO®. The trough carrier is angled to minimize dead volume during aspirate‐dispense operations. This design supports effective and reproducible mixing, enabling better integration and automation of the VI step.

During the low pH VI operation, the custom trough carrier, positioned on the deck, served to contain the diluted pooled protein A eluate following protein A chromatography. Optimized acidification and neutralization ratios, determined through buffer volume screening as previously discussed, were subsequently applied to this pooled material within the trough. To ensure adequate and efficient mixing while concurrently mitigating protein aggregation, various mixing strategies for the trough were evaluated by adjusting both the mixing duration and rpm.

Given the critical nature of minimizing protein aggregation during this process,^10^, ^17^ it is important to consider the potential impact of different mixing methods on aggregate content, as highlighted throughout the literature.^18^ Studies have shown that mechanical stress, a common byproduct of mixing, can induce protein degradation and subvisible particle (SvP) formation, which directly impacts product quality.^19^, ^20^ Specifically, Kiese et al.^18^ demonstrated a notable difference in protein quality outcomes between shaking and stirring methods for mAbs. Shaking primarily led to the formation of soluble aggregates, as detected by SE‐HPLC. In contrast, stirring predominantly resulted in increased turbidity and the formation of visible and subvisible particles. This suggests that the specific type of mechanical agitation employed can dictate the nature of protein aggregates formed. Furthermore, while shear stress alone at high levels may not cause protein denaturation,^21^ its combination with other stress modes can be detrimental. The observations by Kiese et al.^18^ underscore that subtle differences in mixing mechanisms can have profound effects on aggregation pathways.^9^

**FIGURE 5:**
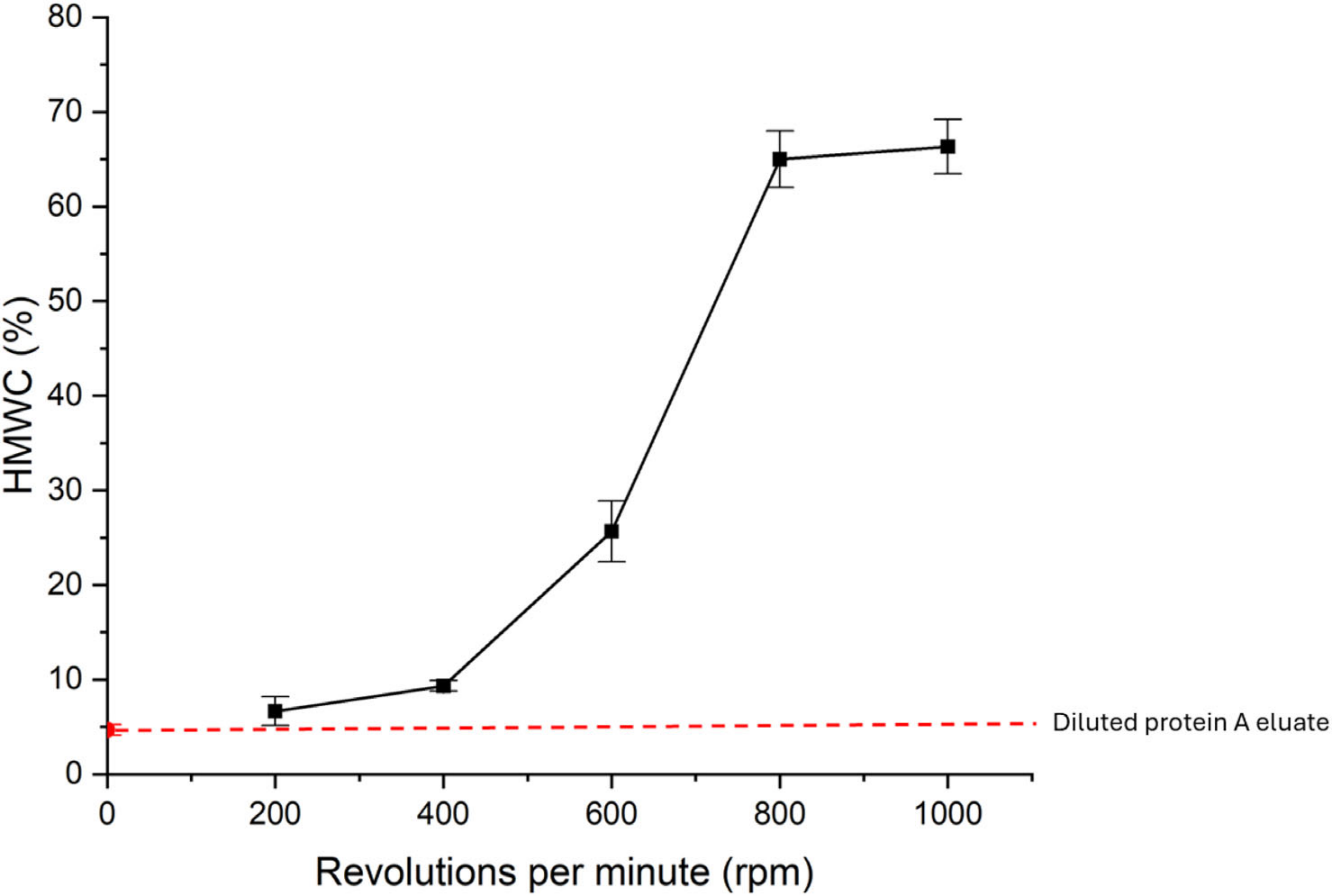
Effect of revolutions per minute (rpm) on aggregate formation during low pH viral inactivation within the 200 mL trough on the BioShake module. High molecular weight components (HMWC) of the protein A eluate were measured after a 60 min low pH (pH 3.5) hold followed by neutralization to pH 5.5. The red dashed reference line indicates the HMWC level of the diluted protein A eluate prior to agitation (0 rpm control). Error bars represent the standard deviation from triplicate runs.

Therefore, when assessing various mixing strategies within the trough, it was crucial to consider not only homogeneity but also the specific mechanical stresses imparted by the chosen mixing action. A strategy that minimizes air‐liquid interfacial stress, which is exacerbated by turbulence and can lead to protein unfolding and aggregation, was sought. Figure 5 presents the effect of the mixing strategy, using the Bioshake module with the custom trough carrier, on the aggregation behavior of the mAb.

Figure 5 illustrates the effect of orbital shaking speed on aggregation within a 200 mL trough during low pH VI. The diluted protein A eluate (0 rpm control) exhibited an HMWC level of approximately 6%, indicated by the red dashed reference line. At 200 rpm, the measured HMWC remained closest to this baseline value, indicating minimal agitation‐induced aggregation under these conditions. Increasing the shaking speed to 400 rpm resulted in a modest rise in HMWC, while agitation at ≥600 rpm caused a pronounced increase in aggregate formation, with HMWC exceeding 25% at 600 rpm and rising sharply to >60% at 800–1000 rpm. This increase is likely due to air entrapment as a result of air‐liquid interfacial stress, enhanced shear‐induced unfolding, and a greater frequency of intermolecular collisions.^19^, ^20^ The plateau observed beyond 800 rpm suggests that aggregation becomes limited by the availability of unfolded or partially unfolded species once a degree of agitation is applied. These findings confirm that maintaining the BioShake at or below 400 rpm is critical to minimizing aggregation. Based on this, the mixing strategy was operated at 200 rpm, which preserves aggregate levels comparable to the diluted protein A eluate baseline.

However, when evaluated using a colourimetric dye test with Bromophenol blue, this yielded an *M*_60_ value of 0.86 ± 0.032 (*n* = 3), indicating that while bulk mixing had improved, it remained insufficient for achieving rapid, uniform pH adjustment. To address this, a mixing strategy, comprising six aspirate‐dispense cycles, was implemented immediately after the addition of the pH‐adjustment buffer and prior to orbital shaking. This combined approach significantly enhanced mixing performance, resulting in an *M*_60_ value of 0.97 ± 0.022 (*n* = 3), comparable to that of the laboratory‐scale mixing method (Table 5). Overall, the employed mixing strategy enabled rapid and homogeneous pH adjustment while effectively minimizing aggregation.

**TABLE 5:** Comparison of mixing efficacy and key operating conditions across the automated mixing method, manual pipette‐based mixing, and laboratory‐scale mixing methods.

Parameter	Automated mixing	Pipette mixing	Laboratory‐scale
Mixing method	Automated mixing using Tecan Freedom EVO® 200	Manual pipette mixing	Mixing in a laboratory‐scale vessel
Protocol	6 aspirate‐dispense cycles with orbital shaking at 200 rpm	3 pipetting steps using a 500 uL pipette	Magnetic stirrer
Liquid volume	40 mL	1 mL	500 mL
Vessel	200 mL trough	96‐well deep well plate	1 L measuring cylinder
Dilution effects	*Minimal* – controlled aspirate and dispense	*Variable* – inconsistent technique	*Low* – due to larger volumes
Repeatability	*High* – defined protocol on an automated system	*Moderate* – user‐dependent	*High* – standardized process
Mixing efficacy (*M*_60_)	0.97 ± 0.022	0.81 ± 0.03	0.97 ± 0.017

### 3.4 Evaluation of pH on cation exchange chromatography performance

**FIGURE 6:**
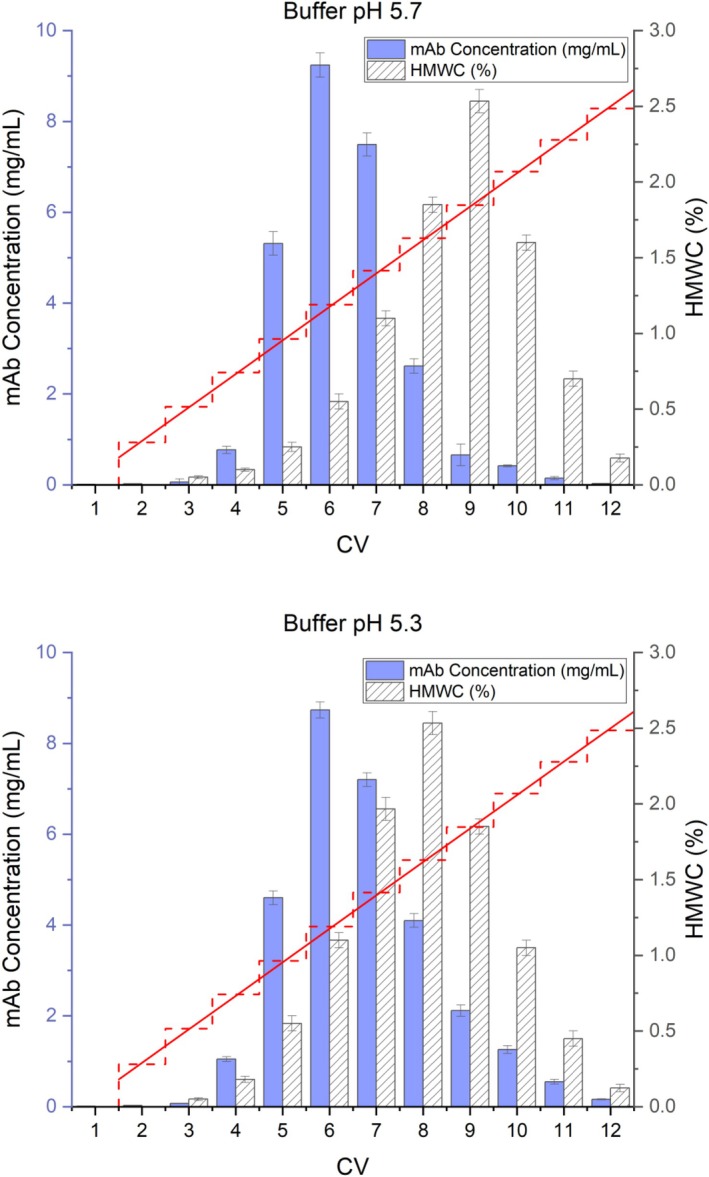
Representative elution profiles showing monoclonal antibody (mAb) concentration and high molecular weight components (HMWC) as a function of column volumes (CV) for cation exchange chromatography elution at buffer pH 5.3 and pH 5.7.

The integrated micro‐scale platform was employed to assess the impact of pH on separation performance during the CEX chromatography operation. Representative CEX elution profiles obtained at pH 5.3 and pH 5.7 are shown in Figure 6 and provide direct insight into the chromatographic behavior underlying differences in monomer recovery and aggregate clearance. These chromatograms highlight how changes in pH, applied within an identical automated workflow, can alter separation performance and product quality attributes. In this study, load adjustment was not conducted. The protein solution loaded onto the CEX column was maintained at a single target condition, ensuring that the incoming material remained constant across all experiments. Therefore, the experimental variable was restricted to the buffer composition. To maintain consistent chromatographic conditions throughout the gradient, the elution buffer was pH‐matched to the respective equilibration buffer for each condition tested. Consequently, the study evaluates the effect of equilibration, and correspondingly elution, buffer pH on separation performance, while holding the product load material constant.

Importantly, the starting material applied to the CEX column contained 4% HMWC, providing a sufficiently challenging impurity load to assess the aggregate clearance capability of the polishing step under each condition.

At pH 5.3, the monomer elution profile is broader, and exhibits pronounced tailing across the conductivity gradient. In parallel, HMWCs elute earlier and overlap with the trailing edge of the monomer peak, indicating reduced chromatographic resolution and increased co‐elution. This behavior suggests that under lower pH conditions, the separation between monomeric and aggregated species is compromised, leading to elevated HMWC levels in the collected product fractions. The broadening of the monomer peak further implies heterogeneous binding interactions, which can complicate pool definition and negatively impact process robustness.

This elution behavior is consistent with the increased net positive charge of the antibody at lower pH. Under these conditions, both monomeric and aggregated species experience stronger electrostatic attraction to the negatively charged CEX resin. While this enhances overall binding, it reduces differential retention between species, thereby diminishing selectivity.^22^, ^23^ As a result, aggregates are insufficiently retained relative to the monomer and elute earlier during the conductivity gradient. The effect is exacerbated as conductivity increases, due to electrostatic screening that weakens charge‐based discrimination between species, further promoting peak overlap and aggregate co‐elution.^24^

In contrast, at pH 5.7, the chromatograms display a sharper, more symmetrical monomer peak with significantly reduced tailing. HMWCs elute earlier in the gradient and are more clearly separated from the monomer fraction, indicating improved resolution and more effective aggregate exclusion. The reduced net positive charge at higher pH weakens overall protein–resin interactions but enhances selectivity between monomeric and aggregated species. Aggregates, which often exhibit altered surface charge distributions and multivalent interaction behavior, appear to retain stronger interactions with the resin relative to monomer under these conditions, resulting in delayed elution and improved clearance.^22^

The improved peak shape and separation observed at pH 5.7 have important implications for process development. Narrower monomer peaks reduce pooling uncertainty, improve yield consistency, and lower the risk including aggregates in the product pool. From a quality perspective, the clearer separation of HMWC demonstrates that adjustments to buffer pH can have a large impact on impurity clearance, underscoring the sensitivity of polishing chromatography to operating conditions.

Critically, these insights were generated using the fully automated, integrated micro‐scale platform, in which the material experienced the complete upstream sequence prior to CEX chromatography. This ensures that the observed chromatographic behavior reflects realistic process intermediates rather than artificially prepared feeds. The ability to generate such mechanistically informative elution profiles in parallel, with minimal material consumption and without manual intervention, demonstrates the value of the platform as a rapid assessment tool for downstream process development.

The platform enables direct visualization of separation behavior, allowing early identification of sub‐optimal conditions through changes in peak shape, resolution, and elution order. This capability is particularly valuable during early development, where rapid screening and informed decision‐making are essential. Overall, the representative chromatograms illustrate how the automated micro‐scale system can be used not only to identify favorable operating conditions but also to understand the underlying chromatographic mechanisms that govern product recovery and aggregate clearance, thereby supporting robust and knowledge‐driven process development.

## 4 CONCLUSIONS AND FUTURE OUTLOOK

This study demonstrates the successful development and integration of an automated, on‐deck pH measurement and feedback control method within a micro‐scale HTPD platform for mAb purification. The method utilizes optical pH sensors immobilized within 96‐well microplates to enable non‐invasive measurement of pH across the acidic range required for low pH VI. Through optimization of measurement conditions, including controlled adjustment of ionic strength, strong agreement between measured and reference pH values was achieved, demonstrating accurate and reproducible performance under process‐relevant conditions.

Implementation of the automated feedback control strategy enabled precise, autonomous adjustment of acid and base additions during both acidification and neutralization phases. This fully walk‐away operation eliminates manual intervention, reduces operator variability, and ensures reproducible attainment of target pH values. Integration of the VI operation with downstream polishing steps, including Sartobind® Q and CEX chromatography, further demonstrated the capability of the platform to support end‐to‐end automated process execution within a single workflow.

The platform was further applied to assess the impact of buffer pH on CEX chromatography performance within an identical automated workflow, demonstrating its capability to evaluate process parameters while maintaining constant load conditions. More broadly, the system functions as a rapid assessment and screening tool, enabling parallel evaluation of multiple conditions and expansion of the accessible experimental space without increasing operator burden. The ability to generate such chromatographic data in parallel, without manual intervention, positions the platform as a powerful rapid assessment and screening tool for downstream process development. By enabling systematic evaluation of operating conditions while holding load material constant, the system expands accessible experimental space and facilitates early identification of sub‐optimal conditions through direct visualization of separation behavior. This capability supports informed decision‐making and accelerates understanding of the mechanistic drivers of product recovery and impurity clearance.

Beyond improved accuracy and automation, the on‐deck pH measurement strategy addresses a critical technological gap that has historically limited full integration of non‐chromatographic unit operations within HTPD platforms. By combining automated sensing, feedback control, and integrated execution of sequential unit operations, the system enhances robustness, reproducibility, and throughput while maintaining alignment with large‐scale process design principles.

Looking forward, this integrated approach establishes a foundation for further expansion toward additional non‐chromatographic operations, including buffer exchange, viral filtration, and formulation development. Incorporation of complementary analytical sensors and advanced control algorithms will enable multi‐parametric monitoring and adaptive decision‐making, advancing the platform toward a comprehensive process analytical technology framework. Overall, this work demonstrates the applicability of the fully integrated micro‐scale platform as a scalable, end‐to‐end automation system capable of accelerating data‐driven process development for mAb downstream production.

## AUTHOR CONTRIBUTIONS

**Sabine Schweisgut:** Investigation; writing – review and editing; data curation. **Nikolas von den Eichen:** Conceptualization; investigation; writing – review and editing; methodology; software; data curation. **Michael Schmitt:** Conceptualization; supervision; formal analysis. **Lars Robbel:** Conceptualization; funding acquisition; writing – review and editing; supervision; formal analysis. **Paras Sharma:** Conceptualization; investigation; writing – original draft; visualization; writing – review and editing; data curation; methodology; formal analysis; project administration. **Daniel G. Bracewell:** Conceptualization; writing – review and editing; funding acquisition; formal analysis; project administration; supervision.

## FUNDING INFORMATION

This work was supported by EPSRC Doctoral Training Partnership (DTP) (EP/T517793/1) and CSL Innovation GmbH.

## CONFLICT OF INTEREST STATEMENT

The authors declare no conflicts of interest.

## Supporting information


**Figure S1.** The relationship between reference pH (x axis) and measured phase shift (y axis) obtained from the fluorescence lifetime‐based optical pH sensor is shown.

## Data Availability

The data that support the findings of this study are available from the corresponding author upon reasonable request.
