# Supplementary material for: On‐deck pH measurement with integrated feedback control on a micro‐scale platform for monoclonal antibody low pH viral inactivation
Source: Biotechnol Prog. 2026 May 5;42(4):e88516. doi: 10.1002/btpr.88516 (PMC13441358; doi:10.1002/btpr.88516)
Supplement: Supplementary file 1 [file BTPR-42-e88516-s001.docx]

*Supplementary Information*

***Figure S1.*** *The relationship between reference pH (x axis) and measured phase shift (y axis) obtained from the fluorescence lifetime-based optical pH sensor is shown. The phase shift (dimensionless, reported in relative units) reflects the change in fluorescence lifetime of the immobilised pH-sensitive dye as a function of proton activity. Blue markers represent experimentally measured calibration points across the tested pH range, while the dashed orange line indicates the fitted sigmoidal calibration model used to convert phase shift values to pH during automated measurements. The characteristic sigmoidal response reflects the acid-base equilibrium behaviour of the sensor dye, with the steep transition region corresponding to the effective dynamic range of the sensor.*
